# Evidence That Cannabis Exposure, Abuse, and Dependence Are Related to Glutamate Metabolism and Glial Function in the Anterior Cingulate Cortex: A ^1^H-Magnetic Resonance Spectroscopy Study

**DOI:** 10.3389/fpsyt.2020.00764

**Published:** 2020-08-20

**Authors:** Jeremy J. Watts, Ranjini Garani, Tania Da Silva, Nittha Lalang, Sofia Chavez, Romina Mizrahi

**Affiliations:** ^1^ Research Imaging Centre, Centre for Addiction and Mental Health, Toronto, ON, Canada; ^2^ Department of Pharmacology & Toxicology, University of Toronto, Toronto, ON, Canada; ^3^ Department of Psychiatry, University of Toronto, Toronto, ON, Canada; ^4^ Campbell Family Mental Health Research Institute, Centre for Addiction and Mental Health, Toronto, ON, Canada

**Keywords:** cannabis, glutamate, imaging, myo-inositol, substance use disorder, magnetic resonance spectroscopy, glial, anterior cingulate cortex

## Abstract

There is evidence that long-term cannabis use is associated with alterations to glutamate neurotransmission and glial function. In this study, 26 long-term cannabis users (males=65.4%) and 47 non-cannabis using healthy controls (males=44.6%) underwent proton magnetic resonance spectroscopy (^1^H-MRS) of the anterior cingulate cortex (ACC) in order to characterize neurometabolite alterations in cannabis users and to examine associations between neurometabolites, cannabis exposure, and cannabis use behaviors. Myo-inositol, a marker of glial function, and glutamate metabolites did not differ between healthy controls and cannabis users or cannabis users who met criteria for DSM5 cannabis use disorder (n=17). Lower myo-inositol, a putative marker of glial function, was related to greater problematic drug use (F_1,22_ = 11.95, p=.002; Cohen’s f=0.59, large effect; Drug Abuse Screening Test) and severity of cannabis dependence (F_1,22_ = 6.61, p=.17; Cohen’s f=0.44, large effect). Further, past-year cannabis exposure exerted different effects on glutamate and glutamate+glutamine in males and females (glutamate: F_1,21_ = 6.31, p=.02; glutamate+glutamine: F_1,21_ = 7.20, p=.014), such that greater past-year cannabis exposure was related to higher concentrations of glutamate metabolites in male cannabis users (glutamate: F_1,14_ = 25.94, p=.00016; Cohen’s f=1.32, large effect; glutamate+glutamine: F_1,14_ = 23.24, p=.00027, Cohen’s f=1.24, large effect) but not in female cannabis users (glutamate: F_1,6_ = 1.37, p=0.78; glutamate+glutamine: F_1,6_ = 0.001, p=.97). The present results extend existing evidence of altered glial function and glutamate metabolism with cannabis use by providing evidence linking problematic drug use behaviors with glial function as measured with myo-inositol and recent chronic cannabis exposure to alterations in glutamate metabolism. This provides novel directions for the interrogation of the impact of cannabis use on brain neurochemistry.

## Introduction

Cannabis is used by some 4% of the global population. The past decade has seen a doubling of high-frequency users (daily or near daily) and the potency of cannabis plant and extracts has increased by 20% since 2014 (1). In parallel with these changes has been an increased burden of problematic cannabis use in adolescents and adults (1, 2). The effects of cannabis use on brain metabolites, and the functional relevance of these effects, however, remain poorly understood.

Delta-9-tetrahydrocannabinol (THC), the primary psychoactive component of cannabis, acts as a partial agonist of the cannabinoid CB1 receptor which is expressed primarily on GABAergic and glutamatergic neurons, but also on other cell types such as astrocytes and glia (3). Preclinical evidence indicate chronic exposure to THC has short- and long-term effects on glutamate neurotransmission and synaptic plasticity (4, 5). Increasing evidence from neuroimaging studies suggest chronic cannabis exposure is also associated with disturbances of glutamate in humans, with reports of reduced glutamate metabolites in striatal, frontal cortical and white matter regions (6–10).

Along with observations of morphological and functional changes to anterior cingulate cortex (ACC) function (11–16), evidence suggests that dysregulation of glutamate metabolism in this brain region and neurotransmission plays a key role in the development and maintenance of substance use disorders (17). Glutamate metabolism is regulated by astrocytes which are responsible for clearance of synaptic glutamate and provide glutamine to neurons (18). Non-neuronal cells such as microglia and astrocytes are not only critical to short- and long-term neuronal function but are themselves affected by exposure to drugs of abuse (18). Indeed, our group recently demonstrated that a mitochondrial marker associated with glial cells is elevated in cannabis users as compared to age-matched healthy controls (19).

Proton magnetic resonance spectroscopy (^1^H-MRS) is a non-invasive brain imaging technique that permits the *in vivo* measurement of neurometabolites including glutamate, glutamine, and myo-inositol. In imaging studies, myo-inositol is commonly considered a marker of astrocyte function, based on early studies with cultured cells showing high concentrations of myo-inositol in astrocytes but absent or very low concentrations in neuronal cell lines (20–23), although some have disputed this claim of cell-type selectivity (20). Myo-inositol levels are increased with neuroimmune activation (24) and in neuroimmune disease (25), traumatic brain injury (26, 27) and mild cognitive impairment (28). In addiction populations, myo-inositol was reported to be elevated in cocaine and alcohol use disorders, but reduced in cannabis users, and available studies with other substances have largely shown mixed results or no changes in this metabolite (29). Nevertheless, despite evidence linking myo-inositol with neuroinflammatory conditions, our understanding of the underlying neurobiology remains incomplete (25, 29, 30).

Reductions in myo-inositol have been reported across multiple brain regions of cannabis users including hippocampus (31), parietal and temporal lobe white matter (8), left thalamus (32), and ACC [(6, 33); but see (34)]. In striatum, myo-inositol was reported to be reduced in male cannabis users (8) but elevated in female cannabis users (10). Overall, the available evidence creates a picture of brain-wide reductions in myo-inositol in cannabis users, with some evidence that some regional effects may differ by sex. Finally, reductions of myo-inositol in thalamus were associated with increased impulsivity (8, 32), suggesting a link between reduced myo-inositol and behaviors associated with higher risk for drug abuse and dependence.

Whereas changes in myo-inositol in cannabis users appear widespread, changes in glutamate may exhibit regional specificity. Glutamate was reduced in the ACC of adolescent cannabis users (6) although this was not observed in adult cannabis users (34). Reduced glutamate metabolites were also reported in the striatum of female (but not male) cannabis users (8). In other regions studied, glutamate metabolites in cannabis users did not differ from healthy controls in hippocampus, parietal lobe, temporal lobe or frontal white matter (8, 10, 31, 35, 36).

The relationship between cannabis exposure, abuse and dependence and neurometabolites is poorly understood. In the ACC, glutamate and myo-inositol were not related to lifetime or past-month cannabis use, urine cannabis metabolites, or age of onset of cannabis use (6, 34). To date, no ^1^H-MRS study in cannabis users has examined how ACC glutamate metabolites or myo-inositol relate to measures of cannabis abuse and dependence.

Based on the observation of lower glutamate metabolites and myo-inositol in ACC, we expect to observe lower glutamate and myo-inositol in the ACC. We also hypothesize that myo-inositol will be negatively associated with cannabis use and measures of cannabis abuse and dependence, and that glutamate will be negatively associated with past-year cannabis use.

## Materials and Methods

### Participants

Twenty-six cannabis users and forty-seven healthy control participants completed ^1^H-MRS scans. All participants had no history of psychiatric illness including substance use disorders (except nicotine or caffeine in all participants, or cannabis in cannabis users) as determined by the SCID, and had no family history of psychotic disorders. Participants were excluded for past or present alcohol abuse or dependence in the past 6 months. Current use of alcohol was permitted. Participants were excluded if they were pregnant or breastfeeding, or had medical illness or metal implants precluding magnetic resonance imaging (MRI). Cannabis users were invited to participate if they currently used cannabis at least 4 days per week, had been using at that or higher frequency for at least one year, and tested positive for cannabis use at the baseline study visit. All participants were required to test negative for drugs of abuse on a urine drug screen, except for cannabis in cannabis users. The urine immunoassay tested for ethanol, methadone, benzodiazepines, cannabinoids, opiates, and cocaine metabolites (benzoylecgonine).

Drug use history was assessed by a semi-structured interview, including standard questions and participant-specific drug history, established using a natural history interview approach (5) covering the period from the first cannabis use to the date of the interview. Standardized questions include age of first use, route of administration, unit dose, frequency, and dose used during the past year, motivation for use and problems caused by use. Current use was confirmed using a urine drug screen. Problematic drug use was assessed using the Drug Abuse Screening Test (DAST) and cannabis dependence was assessed using the Severity of Dependence scale for cannabis (SDS).

This study was approved by the Research Ethics Board at the Centre for Addiction and Mental Health (CAMH). All participants provided written informed consent after receiving a description of all study procedures.

### ^1^H-Magnetic Resonance Spectroscopy

#### MRS Acquisition and Analysis

^1^H-MRS scans were performed at the CAMH Research Imaging Centre (Toronto, Canada) using a 3T General Electric Discovery MR750 scanner (Milwaukee, WI, USA) equipped with an 8-channel head coil. Head motion was minimized by positioning each subject at the center of the head coil with soft restraint padding around the head and tape strapped across the forehead. T1-weighted fast spoiled-gradient-echo 3-dimensional sagittal acquisition scans were acquired for each participant (FSPGR sequence, TE=3.0 ms, TR=6.7 ms, TI=650 ms, flip angle=8°, FOV=28 cm, acquisition matrix 256 × 256 matrix, slice thickness=0.9 mm).

**Figure 1:**
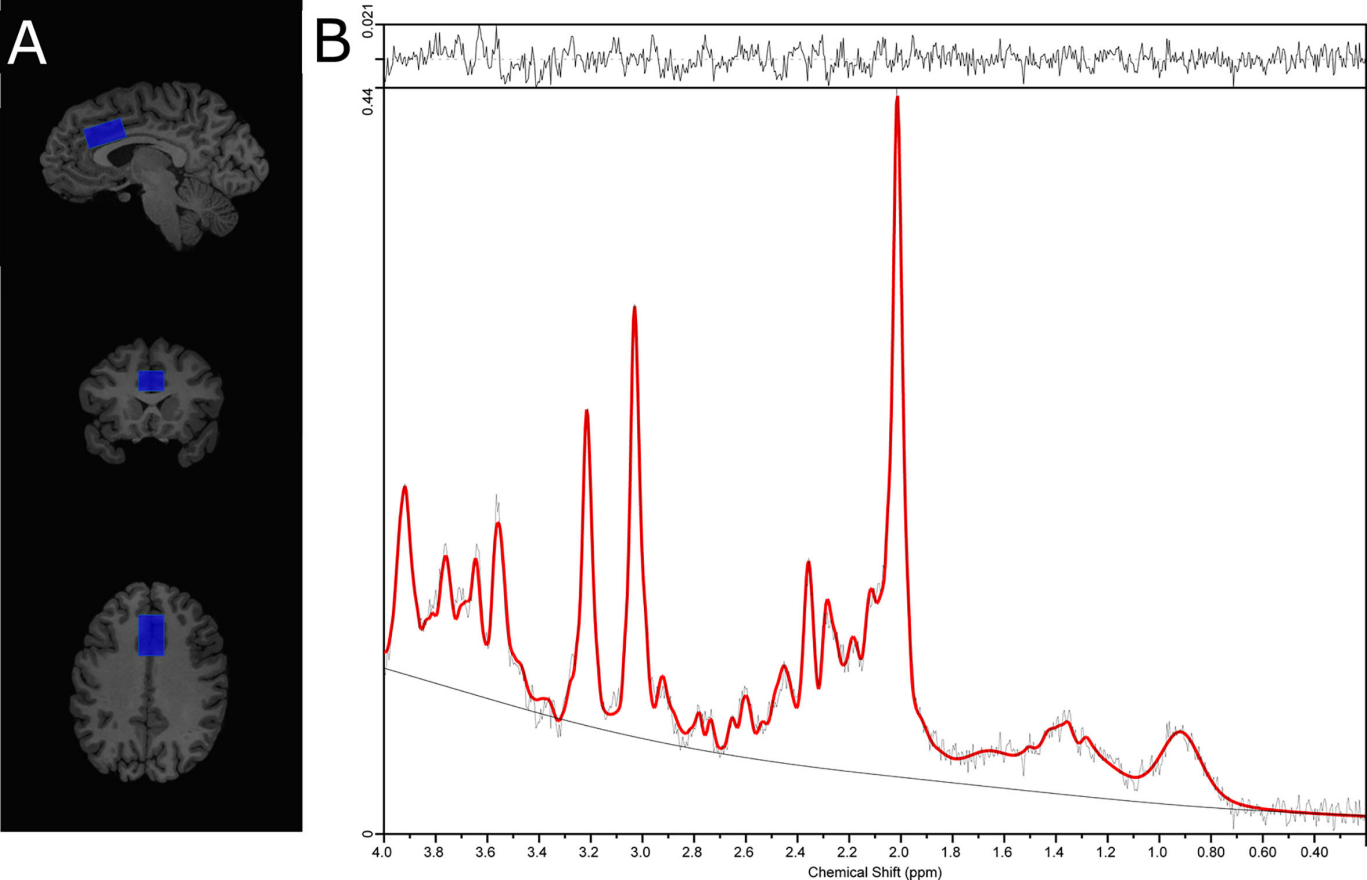
**(A)** Placement of the supragenual anterior cingulate cortex (ACC) voxel and, **(B)** ACC ^1^H-MRS spectra from a representative subject.

Single voxel ^1^H-MRS spectra were obtained using the standard GE Proton Brain Examination (PROBE) sequence with point-resolved spectroscopy (PRESS sequence, TE=35 ms, TR=2000 ms, number of excitations=8, bandwidth=5,000 Hz, 4,096 data points used, 128 water-suppressed, and 16 water-unsuppressed averages. For each participant, the voxel (30 x 20 x 15 mm APxRLxSI) was carefully positioned on the bilateral supragenual anterior cingulate cortex (ACC; **Figure 1**). The voxel placement was independently confirmed during placement and again during analysis by a trainer independent rater. For each region, the signal over the voxel was shimmed to achieve a linewidth of 12 Hz or less, measured from the unsuppressed water signal in the voxel.

#### Data Processing and Analysis

MRS data were analyzed with LC Model version 6.3-0E (37), using a standard basis set of metabolites (listed below). Spectra fits yielded relative quantification of metabolite concentration levels. This was achieved by normalizing the metabolite fits to the unsuppressed water signal, corrected for fraction of water in each compartment (grey matter (GM), white matter (WM) and cerebrospinal fluid (CSF)). Neurometabolite quantities were thus expressed in institutional units (IU) that could be compared across individuals regardless of variations in CSF contributions to the voxel signal. A field-appropriate basis set with TE = 35 ms was used, and contained L-alanine, aspartate, Cr, Cr methylene group, γ-aminobutyric acid, glucose, glutamate, glutamine, glutathione, glycerophosphocholine, L-lactate, myo-inositol, NAA, N-acetylaspartylglutamate, phosphocholine, phosphocreatine, scyllo-inositol, and taurine, as well as the following lipids (Lip) and macromolecules (MM): Lip09, Lip13a, Lip13b, Lip20, MM09, MM12, MM14, MM17, and MM20.

All scans included in statistical analyses met ^1^H-MRS quality control cutoffs (full-width at half-maximum (FWHM) ≤0.1, signal-to-noise ratio (SNR) ≥10, Cramér-Rao lower bounds ≤15%), scans failing to meet QC cutoffs or meeting criteria for rejecting analyses described in the LC Model manual were removed from the analyses (38).

#### Voxel Tissue Heterogeneity

T1-weighted MRI scans used for voxel localization were segmented into GM, WM, and CSF using FSL5.0 (FAST; FMRIB Analysis Group, Oxford University, UK). Voxel location, orientation and size information obtained from spectra file headers were used to generate binary masks in the same matrix as the T1 image (Gannet 2.1, (39). Binary voxel masks were applied to segmented T1 images in order to determine percentages of GM, WM, and CSF within the ^1^H-MRS voxel (36).

The water-scaled metabolite concentrations were corrected for voxel tissue composition as follows. To get the observed metabolite concentration (not corrected for metabolite relaxation times), relative to a fully relaxed water concentration in tissue, [M], the volume fractions, water relaxation times (T1, T2) and water concentrations of the three compartments: WM, GM and CSF, must be taken into account per (40). Values for relaxation times at 3T were based on (41–44). To reconcile the operation already performed by LC Model to give water-scaled data (i.e., [M]*_WS_*), we performed the following:

$$\left[M\right]=\frac{{\left[M\right]}_{WS}[\left(f_{CSF}*55556*R_{CSF}\right)+\left(f_{GM}*43300*R_{GM}\right)+\left(f_{WM}*35880*R_{WM}\right)}{\left(0.7*35880*\left(1-f_{CSF}\right)\right)}$$

Where, $Ri=\left(1-e^{\left(-\frac{TR}{T1i}\right)}\right)*e^{\left(-\frac{TE}{T2i}\right)}$

For i=WM, GM, and CSF

and where 0.7 * 35880 is used to account for the assumptions used by LC model (i.e., WCONC=35,880 and $ATT20=0.7=e^{\left(-\frac{30}{80}\right)}$).

### Statistical Analysis

Analyses were performed using SPSS (version 24.0; IBM Corporation, Armonk, NY, USA). Group differences in sample characteristics were tested using t-tests or chi-square tests for continuous and categorical variables, respectively (**Table 1**).

The effect of group on ^1^H-MRS neurometabolites were tested using a general linear model including sex and creatine (creatine+phosphocreatine) as covariates. Creatine is highly correlated with other metabolites of interest in the ^1^H-MRS spectrum, therefore creatine was included as a covariate in all analyses (45). Previous ^1^H-MRS studies in cannabis users indicated that including sex in their model affected their results with glutamate metabolites and/or myo-inositol (6, 10, 31, 34), therefore all statistical models included sex as a factor.

The effects of problematic drug use, severity of cannabis dependence and past-year cannabis exposure on ^1^H-MRS neurometabolites were tested using a general linear model including sex and creatine (creatine+phosphocreatine) as covariates. Non-significant interaction terms were removed from the model. Significant interaction terms were followed up using a general linear model. Each variable was tested with myo-inositol, glutamate and glutamate+glutamine, therefore a statement of statistical correction (3 metabolites) accompanies reporting of results.

## Results

### Demographics and ^1^H-MRS Scan Characteristics

In total, 54 healthy control participants and 26 cannabis users underwent ACC ^1^H-MRS scans. Data for seven healthy control participants did not meet quality control standards (FWHM>.1), therefore the present analysis includes 47 healthy control participants and 26 cannabis users.

Healthy controls and cannabis users did not differ significantly in age or sex, however there were more tobacco smokers in the cannabis user group (**Table 1**). The linewidth (FWHM), signal-to-noise ratio (SNR) and Cramér-Rao lower bounds for all metabolites were comparable in healthy controls and cannabis users (**Table 1**). Tissue grey matter fraction was lower in the cannabis user group (F_1,69_ = 4.82, p=.031), but groups did not differ in white matter (F_1,69_ = 2.69, p=.11) or CSF fractions (F_1,69_ = 0.44, p=.56).

**Table 1:** Sample characteristics (mean ± SD) [range].

		Healthy controls	Cannabis users	t/χ^2^	p
***Sample size***		47	26		
***Age (years)± SD***		23.38 ± 3.88 [18-37]	23.76 ± 4.20 [18-35]	0.15	.69
***Gender***	Male	21	17	2.88	.09
	Female	26	9		
***Current drug use***	Tobacco	0	4	7.65	.001
	Other drugs of abuse	0	0		
	Cannabis	0	26		
***Cannabis exposure and measures of drug abuse and dependence***	Age at first use (years)		16.53 ± 3.39 [12-29]		
	Age at regular use (years)		19.23 ± 4.27 [14-34]		
	Estimated lifetime cannabis use (grams)		2284 ± 1684		
	Estimated past-year cannabis use (grams)		455 ± 256		
	Current daily use (grams)		1.39 ± 0.82		
	CUD/no CUD		17/9		
	SDS		2.77 ± 2.18		
	DAST		4.42 ± 3.40		
***^1^H-MRS***					
	FWHM	0.036 ± 0.011	0.036 ± 0.0094	0.041	.84
	SNR	25.79 ± 4.29	25.58 ± 4.13	0.041	.84

CUD, cannabis use disorder; SDS, severity of dependence scale; DAST, drug abuse screening test; FWHM, full-width at half-maximum; SNR, signal-to-noise ratio.

^1^H-MRS creatine is strongly associated with other neurometabolites (myo-inositol: F_1,70 _= 24.71, p=4.55x10^-6^, glutamate: F_1,70 _= 10.68, p=.0017 and glutamate+glutamine: F_1,70_ = 16.45, p=.00013) as previously reported (45).

Consistent with previous literature, ^1^H-MRS metabolites of interest differed significantly by sex (creatine: F_1,70_ = 4.35, p=.041; myo-inositol: F_1,70_ = 4.29, p=.042; glutamate: F_1,70 _= 6.06, p=.016; glutamate+glutamine: F_1,70_ = 21.11, p=.000019) therefore sex was included in all models.

### ^1^H-MRS Neurometabolites Do Not Differ Between Healthy Controls and Cannabis Users

**Figure 2:**
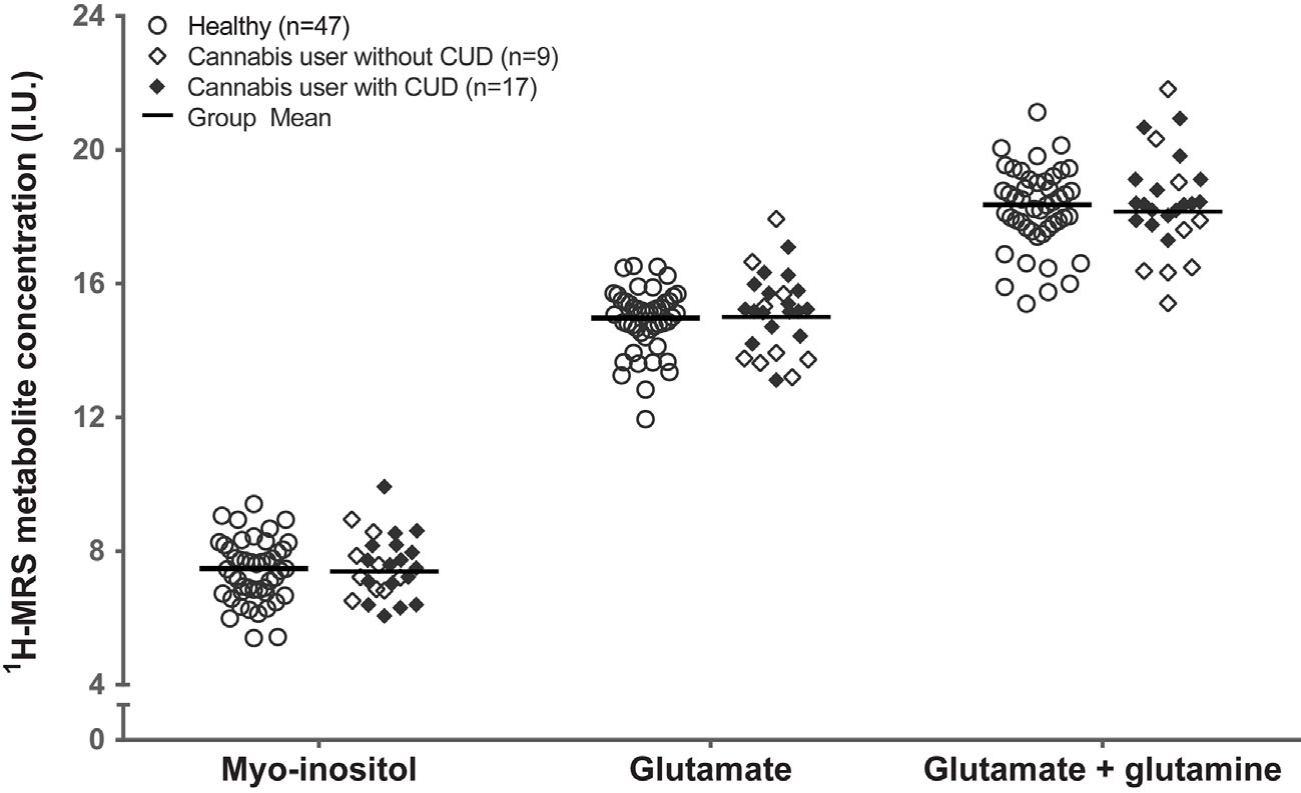
Groupwise scatter of ^1^H-MRS metabolites in healthy control participants and in cannabis users with and without cannabis use disorder. Group mean reflects estimated marginal mean for the healthy control participants (n=47) and cannabis users (n=26), controlling for sex and creatine. CUD, cannabis use disorder; I.U., institutional units.

Controlling for sex, healthy controls and cannabis users did not differ significantly in creatine (F_1,69_ = 1.48, p=.23; **Figure 2**). Controlling for sex and creatine, healthy controls, and cannabis users did not differ in myo-inositol (F_1,69_ = 0.18, p=.67; myo-inositol), glutamate (F_1,69_ = 0.018, p=.89), or glutamate+glutamine (F_1,69_ = 0.56, p=.46). These results remained unchanged after controlling for tobacco use (myo-inositol: F_1,68_ = 0.62, p=.43; glutamate: F_1,68_ = 0.04, p=.85; glutamate+glutamine: F_1,68_ = 0.29, p=.59).

### Myo-Inositol Is Related to Severity of Drug Abuse and Dependence

**Figure 3:**
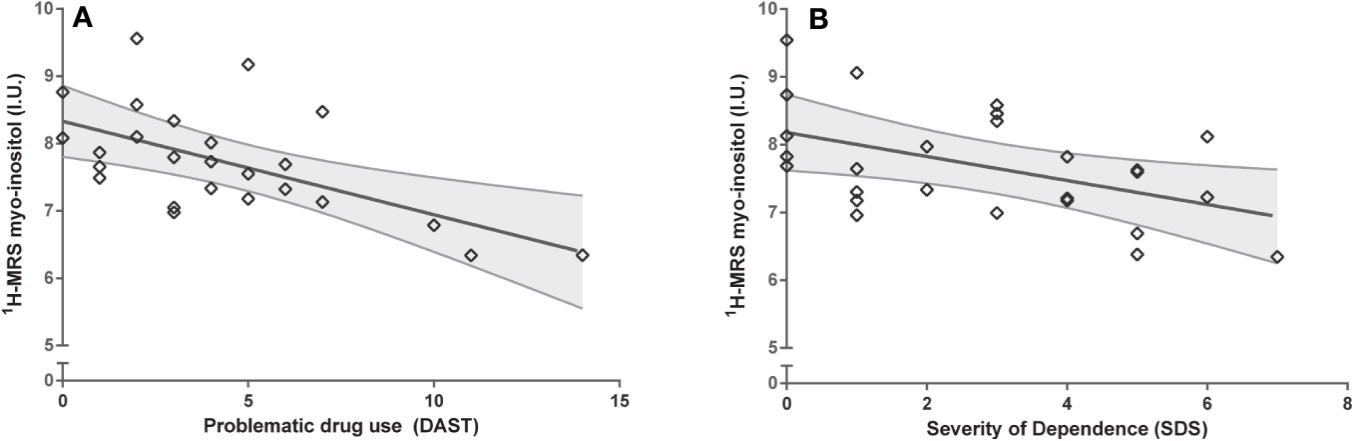
Associations between myo-inositol and **(A)** problematic drug use behaviors (DAST) and **(B)** Severity of cannabis dependence (SDS). Values adjusted for creatine and sex. I.U., institutional units.

Controlling for sex and creatine, myo-inositol was significantly associated with problematic drug use behaviors (DAST), such that cannabis users with the most problematic drug use exhibited the lowest levels of myo-inositol (F_1,22_ = 11.95, p=.002, Cohen’s f=0.59, large effect; **Figure 3**). Similarly, controlling for sex and creatine, myo-inositol was significantly associated with severity of cannabis dependence score (SDS) (F_1,22_ = 6.61, p=.017, Cohen’s f=0.44, large effect; **Figure 3**). The associations of myo-inositol with problematic drug use behaviors and severity of dependence remained significant after controlling for tobacco use (DAST: F_1,21_ = 7.95, p=.005; SDS: F_1,21_ = 6.61, p=.018). The association of myo-inositol with problematic drug use behaviors remains significant after correction for multiple testing but the association with severity of dependence does not survive correction.

In contrast to results with myo-inositol, glutamate and glutamate+glutamine were not significantly associated with problematic drug use behaviors (DAST; glutamate: F_1,22_ = 0.36, p=.55; or glutamate+glutamine: F_1,22_ = 2.15, p=.16) or severity of dependence (SDS; glutamate: F_1,22_ = 0.01, p=.94; or glutamate+glutamine: F_1,22_ = 0.17, p=.90).

### Glutamate Metabolites Are Related to Past-Year Cannabis Exposure

**Figure 4:**
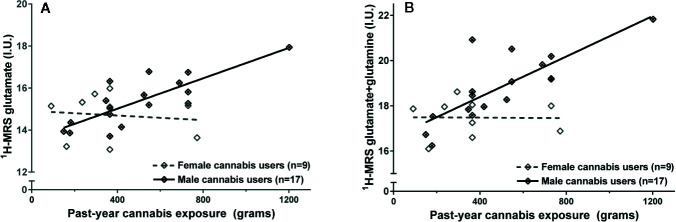
Greater past-year cannabis exposure is associated with higher **(A)** glutamate and **(B)** glutamate+glutamine in male but not in female cannabis users. Values adjusted for creatine and sex. I.U., institutional units.

Glutamate and glutamate+glutamine exhibited a significant interaction with sex on past-year cannabis exposure (glutamate: F_1,21_ = 6.31, p=.02; glutamate+glutamine: F_1,21_ = 7.20, p=.014), such that greater past-year cannabis exposure was related to higher concentrations of glutamate metabolites in male cannabis users (glutamate: F_1,14_ = 25.94, p=.000016; Cohen’s f=1.32, large effect; glutamate+glutamine: F_1,14_ = 23.24, p=.00027, Cohen’s f=1.24, large effect; **Figure 4**) but not female cannabis users (glutamate: F_1,6_ = 0.082, p=.78; glutamate+glutamine: F_1,6_ = 0.001, p=0.97; **Figure 4**).

The outcome of these tests were not meaningfully altered after controlling for tobacco use (males: glutamate: F_1,13_ = 23.84, p=.00003; glutamate+glutamine: F_1,13_ = 21.19, p=.0005; female cannabis users: glutamate: F_1,5_ = 0.06, p=.81; glutamate+glutamine: F_1,5_ = 0.001, p=.98). The associations between glutamate metabolites and past-year cannabis exposure survives correction for multiple tests.

These effects were not driven by the male cannabis user with particularly high past-year cannabis use (**Figure 4**), as the results did not change if this high-exposure participant was excluded from the analysis (interaction: glutamate: p=.041; glutamate+glutamine: p=.025; association with past-year cannabis use: glutamate: p=.004; glutamate+glutamine: p=.004).

Finally, in contrast to results with glutamate metabolites, myo-inositol was not significantly associated with past-year cannabis exposure (F_1,22 _= 0.47, p=.50).

## Discussion

In this study, we observed that young adult cannabis users do not differ in myo-inositol or glutamate metabolites in the ACC relative to demographically matched healthy control participants. Cannabis users with greater drug abuse severity had lower levels of myo-inositol. Greater past-year cannabis exposure was strongly associated with higher levels of glutamate metabolites in the ACC, and this effect was observed in male but not female cannabis users.

To date, reductions of glutamate metabolites and myo-inositol levels in ACC were observed in adolescent (16–19 years) (6) but not in ACC of young adult (18–39 years) cannabis users (34). The adolescent and young adult studies differ also in cannabis use history, ^1^H-MRS acquisition, and voxel size and placement. An expanding body of evidence suggests that adolescents and adults may be differently affected by short and long-term cannabis exposure (4, 46). Alternatively, given the placement and size of the larger voxel in that study, the reductions in glutamate metabolites observed in adolescent cannabis users may reflect changes in medial prefrontal cortex rather than ACC (7). Therefore, further work is needed in order to disentangle the contributions of age and brain region to cannabis-related alterations of brain metabolites.

Lower ACC myo-inositol was associated with greater drug abuse severity. Drug abuse behaviors (e.g, persisting in drug use despite problems caused, inability to limit intake) are often associated with impairments in self-control and impulsivity, which are central functions of the ACC (47). In young adults (21 ± 3.6 years) with cannabis dependence, lower myo-inositol across parietal, temporal, frontal and subcortical regions was associated with greater impulsivity (8, 32). Altogether, results support an association of drug abuse behaviors with myo-inositol in the ACC (8).

Reduced myo-inositol may reflect disturbances of astrocyte function in the ACC of cannabis users. Astrocytes are in contact with hundreds to thousands of synapses, positioning them to regulate both neuroimmune activity and neurotransmission across large areas (18). In animals, astrocytic CB1 receptors contribute to THC-induced memory deficits in the hippocampus, but this has not been studied in the ACC (48, 49). Finally, a disturbance of astrocyte function could be related to changes to glutamate metabolism, however such interactions are not well understood in humans (18, 31).

Male cannabis users with greater past-year cannabis exposure had higher levels of ACC glutamate metabolites. The direction of this effect is unexpected, given reports of lower, but not higher glutamate metabolites in cannabis users (6, 8). The relationship between glutamate and cannabis use history has been largely unexplored. Of eight ^1^H-MRS studies in cannabis users, only three reported testing glutamate metabolites with measures of recent and long-term cannabis exposure (6–10, 31, 34, 35). Glutamate metabolites in mPFC or ACC were not significantly associated with cannabis metabolites, current cannabis consumption, or lifetime cannabis exposure (6, 7, 34). Studies with longitudinal or drug-challenge designs, are needed better understand the nature of the relationship between cannabis exposure and glutamate.

Past-year cannabis use was associated with increased glutamate metabolites in male but not female cannabis users. Few human imaging studies have examined sex differences in glutamate metabolites in cannabis users, or in response to acute THC/cannabis challenge. One study reported that striatal glutamate+glutamine/creatine was reduced in female but not male daily cannabis users (10). In contrast to sex differences in neurometabolic disturbances in cannabis users, a rich and expanding literature describes sex differences in behavioral, neural and long-term neurophysiological effects of cannabis use (50, 51). Future imaging studies should emphasize samples balanced for sex, in order to identify and characterize neurometabolic consequences of cannabis use for males and females (50, 51).

Strengths of the present study include measurement of past and recent cannabis use history, and measures of cannabis abuse and dependence (52, 53). The present sample also included cannabis users with and without cannabis use disorder, permitting dimensional analysis of these variables across diagnostic categories. Finally, ^1^H-MRS measurements were corrected for tissue segmentation as well as the water relaxation times and water concentrations of GM, WM, and CSF.

The present study also has limitations that should be considered. Due to the cross-sectional design, the present results cannot establish a causal relationship between neurometabolites and cannabis abuse, dependence, or exposure. As with most ^1^H-MRS studies in cannabis users, participants in the present study were not treatment-seeking. This study included cannabis users with and without cannabis use disorder, which provided wide variation in problematic drug use behaviors and cannabis exposure, however this may have reduced the ability of this study to observe group differences in metabolites. Finally, the resonances of glutamate and glutamine cannot be reliably separated using the standard short-TE PRESS acquisition at 3T, so we cannot make separate conclusions about these metabolites. Lastly, although myo-inositol is routinely interpreted as an astrocyte-specific marker, some have disputed this claim (20).

The THC and CBD content of the cannabis smoked by users was not measured, therefore our estimates of cannabis use-occasions may not directly translate to THC dose. Although current cannabis use was confirmed by urine drug screens, we did not measure cannabinoids in hair, which would help to verify self-reported cannabis use history. Moreover, there is a need for increased standardization of measures of cannabis exposure, cannabis use characteristics and reporting in future studies in order to facilitate comparison of results across studies (54).

Finally, the present study tested the statistical association of three metabolites with three secondary outcome measures, and although the associations between myo-inositol and drug abuse beahviors and between glutamate, glutamate+glutamine and past-year cannabis use survive statistical correction, the number of tests nevertheless increases the possibility of false positive results.

### Conclusions

Young adult cannabis users do not differ in myo-inositol or glutamate metabolites in the ACC relative to demographically matched healthy young adult control participants. Cannabis users with greater severity of drug abuse behaviors had lower levels of myo-inositol. Greater past-year cannabis exposure was strongly associated with higher levels of glutamate metabolites in the ACC, and this effect was observed in male but not female cannabis users. This study demonstrates the importance of examining measures of drug abuse and dependence in addition to drug exposure in studies of neurometabolites in cannabis users and substance use disorders more generally. Studies with longitudinal designs are needed in order to establish causal relationships between neurometabolites, cannabis exposure and cannabis abuse and dependence.

## Data Availability Statement

The raw data supporting the conclusions of this article will be made available by the authors, without undue reservation.

## Ethics Statement

This study was approved by the Research Ethics Board at the Centre for Addiction and Mental Health (CAMH). All participants provided written informed consent after receiving a description of all study procedures.

## Author Contributions

JW, RG, and RM wrote the manuscript. JW, TS, and NL collected participant data. SC and JW conducted analysis of ^1^H-MRS data and image processing. JW and RG conducted the statistical analysis. RM designed the study and provided medical and scientific oversight. All authors contributed to the article and approved the submitted version.

## Funding

This work was partially supported by a NARSAD Independent Investigator’s Grant (21977) and grants from the National Institute of Mental Health (NIMH) to RM (R21MH103717 and R01MH113564).

Funding bodies had no involvement in the conduct, data analysis, interpretation or reporting of this study.

## Conflict of Interest

RM has received travel support and speaker fees from Janssen Inc and consulting fees from Otsuka-Lundbeck Canada.

The remaining authors declare that the research was conducted in the absence of any commercial or financial relationships that could be construed as a potential conflict of interest.
